# Progression of cardiac allograft vasculopathy assessed by serial three-vessel quantitative coronary angiography

**DOI:** 10.1371/journal.pone.0202950

**Published:** 2018-08-27

**Authors:** Christian Zanchin, Kyohei Yamaji, Carolin Rogge, Dorothea Lesche, Thomas Zanchin, Yasushi Ueki, Stephan Windecker, Paul Mohacsi, Lorenz Räber, Vilborg Sigurdardottir

**Affiliations:** 1 Department of Cardiology, Bern University Hospital, Bern, Switzerland; 2 Institute of Clinical Chemistry, Bern University Hospital, Bern, Switzerland; Beijing Key Laboratory of Diabetes Prevention and Research, CHINA

## Abstract

**Background:**

The purpose of the present study was to assess the short- and long-term progression of cardiac allograft vasculopathy (CAV) using serial 3-vessel quantitative coronary angiography (QCA).

**Methods:**

CAV progression was assessed using serial 3-vessel QCA analysis at baseline, 1-year and long-term angiographic follow-up (8.5±3.7 years) after heart transplantation. The change in minimal lumen diameter (MLD) and percent diameter stenosis (%DS) was serially assessed within matched segments. Patients were graded according to the ISHLT-CAV classification and grouped as ISHLT-CAV_0_ and ISHLT-CAV_1-3_. The primary endpoint was mean change in MLD and %DS.

**Results:**

A total of 41 patients and 520 matched segments were available for serial 3-vessel QCA. Overall, MLD decreased non-significantly from baseline to 1-year follow-up and significantly from 1-year to the long-term angiographic follow-up (Δ-0.08mm/year [95%CI -0.11 to -0.05], P<0.001). %DS increased significantly from baseline to 1-year (Δ+0.96%/year [95%CI 0.04 to 1.88], P = 0.041) and from 1-year to long-term angiographic follow-up (Δ+0.61%/year [95%CI 0.33 to 0.88], P<0.001). ISHLT-CAV_1-3_ at 1 year and at long-term angiographic follow-up was observed in 22% and 61%, respectively. Between baseline and long-term angiographic follow-up, a significant reduction in MLD was observed within both groups without a significant difference in the reduction between the two groups (ISHLT-CAV_0_: median -0.49mm [IQR -0.54 to -0.43] vs. ISHLT-CAV_1-3_: median -0.40mm [IQR -0.44 to -0.35], P = 0.4).

**Conclusion:**

The current data suggest that QCA can’t predict CAV beyond 1 year, but, QCA affirmed that CAV progresses to a similar extent in patients who do not develop visual CAV during long-term follow-up.

## Background

Cardiac allograft vasculopathy (CAV) has become one of the most important cause of long-term mortality after heart transplantation (HTx) [1]. CAV is characterized by a concentric and diffuse proliferation of the coronary arterial intima, resulting in thickening and progressive luminal narrowing [2]. Because the modification in immunosuppressive therapy may delay or even cause CAV regression, the early detection of CAV is of high clinical interest [3–7].

The current guidelines recommend annual or biannual coronary angiography for detection and surveillance of CAV [5]. CAV is classified on a visual assessment of coronary angiographic findings and graft function according to the International Society for Heart and Lung Transplantation (ISHLT) classification [5]. When using the ISHLT-classification, the incidence of CAV is approximately 8% at 1 year, 30% at 5 years, and 50% at 10 years [1]. However, concern remains in the ability of coronary angiography to detect accurately the early stages and progression of CAV [8]. Intravascular ultrasound (IVUS) studies have found a coronary intimal thickening in up to 80% of patients already during the first year after HTx without angiographic signs of CAV and angiographically silent progression of CAV predicts long-term morbidity and mortality after cardiac transplantation [9,10].

Quantitative coronary angiography (QCA) has been used for many years in clinical research to assess the luminal diameter of arteries. It can readily survey the entire coronary vascular system on the coronary angiographies. While QCA makes quantification of coronary atherosclerosis progression possible [11,12], no studies with serial 3-vessel QCA have been conducted to determine CAV progression. Therefore, the aim of this study was to assess CAV progression by using serial 3-vessel QCA at baseline, at 1 year, and at long-term angiographic follow-up after HTx.

## Methods

### Study population

**Fig 1:**
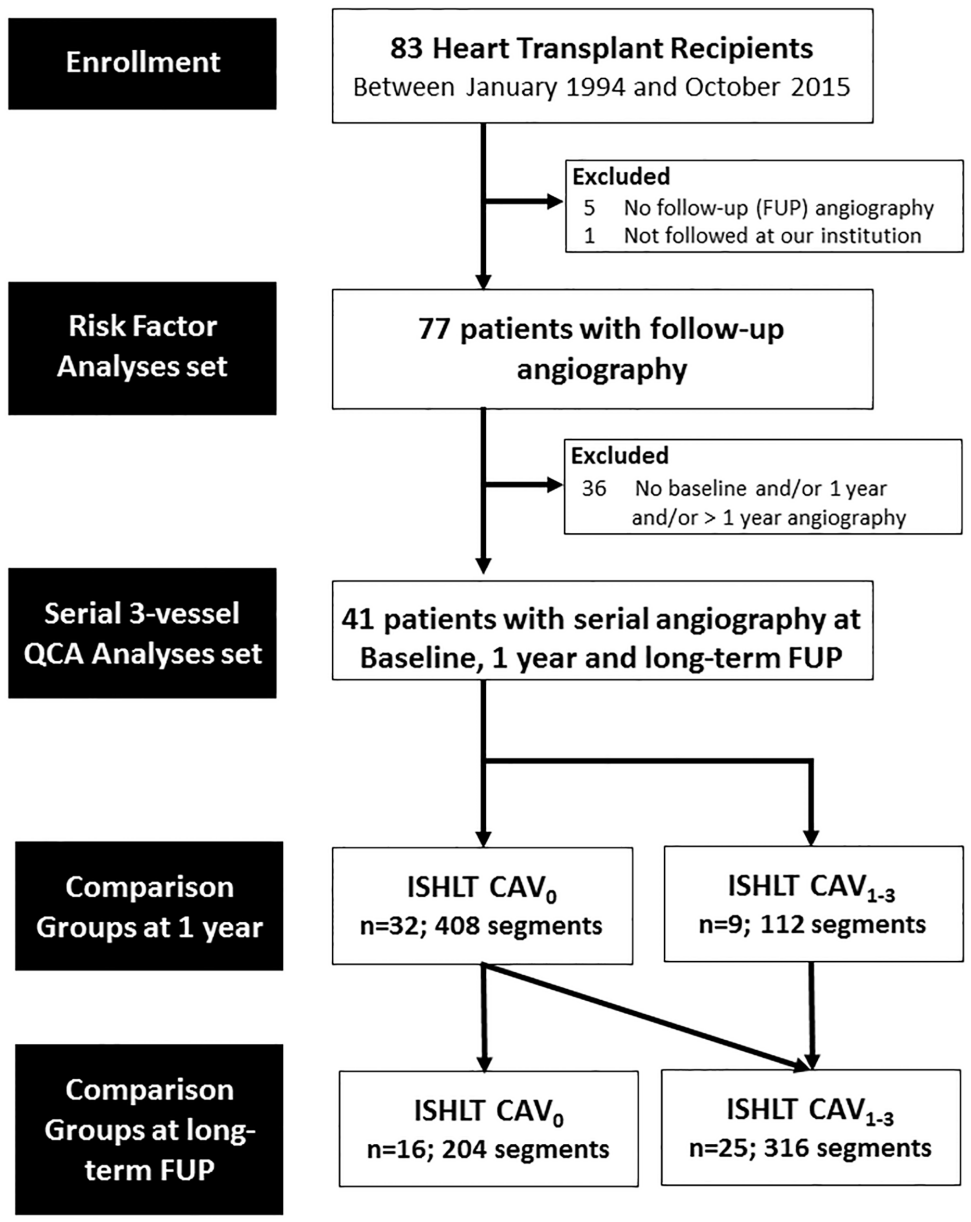
Flow chart. CAV indicates cardiac allograft vasculopathy; FUP, follow-up; QCA, quantitative coronary angiography; ISHLT, International Society of Heart and Lung Transplantation.

This is a retrospective observational cohort study including 83 HTx recipients who underwent heart transplantation between January 1994 and October 2015 at the University Hospital of Bern. Follow-up angiography 1 year after HTx was available in 77 patients and was used to assess CAV risk factors. For the purpose of the serial 3-vessel QCA analyses, we excluded patients without baseline angiography, 1 year follow-up angiography, or long-term follow-up angiography (n = 36). Finally, we enrolled 41 patients in the serial 3-vessel QCA analyses set. A detailed patient flow is shown in Fig 1.

The study complied with the Declaration of Helsinki and the study was approved by the Cantonal Ethics Committee of Bern (CEC 186/14). All patients provided written informed consent. None of the transplant donors were from a vulnerable population and all donors or next of kin provided written informed consent that was freely given.

### Coronary angiography assessment

CAV was classified by a retrospective review of all coronary angiographic and echocardiographic studies at 1 year, and at latest angiographic follow-up after HTx, based on the ISHLT guidelines [5] as follows: ISHLT-CAV_0_ indicates no visually detectable angiographic lesion; ISHLT-CAV_1_ (mild) indicates angiographic left main <50%, or primary vessel with a maximum lesion of <70%, or any branch stenosis <70% (including diffuse narrowing) without allograft dysfunction; ISHLT-CAV_2_ (moderate) indicates angiographic left main <50%, a single primary vessel ≥ 70%, or isolated branch stenosis ≥ 70% in branches of 2 systems, without allograft dysfunction; and ISHLT-CAV_3_ (severe) indicates angiographic left main ≥ 50%, or two or more primary vessels with ≥ 70% stenosis, or isolated branch stenosis ≥ 70% in all 3 systems, or ISHLT-CAV_1_ or ISHLT-CAV_2_ with allograft dysfunction (defined as left ventricular ejection fraction ≤ 45% with the presence of regional wall motion abnormalities). For the current analysis, we defined the presence of CAV as a status ≥ ISHLT-CAV_1_ and we grouped the study population in ISHLT-CAV_0_ and ISHLT-CAV_1-3_. We conducted follow-up and angiographic assessment until April 2016.

### Quantitative coronary angiography assessment

**Fig 2:**
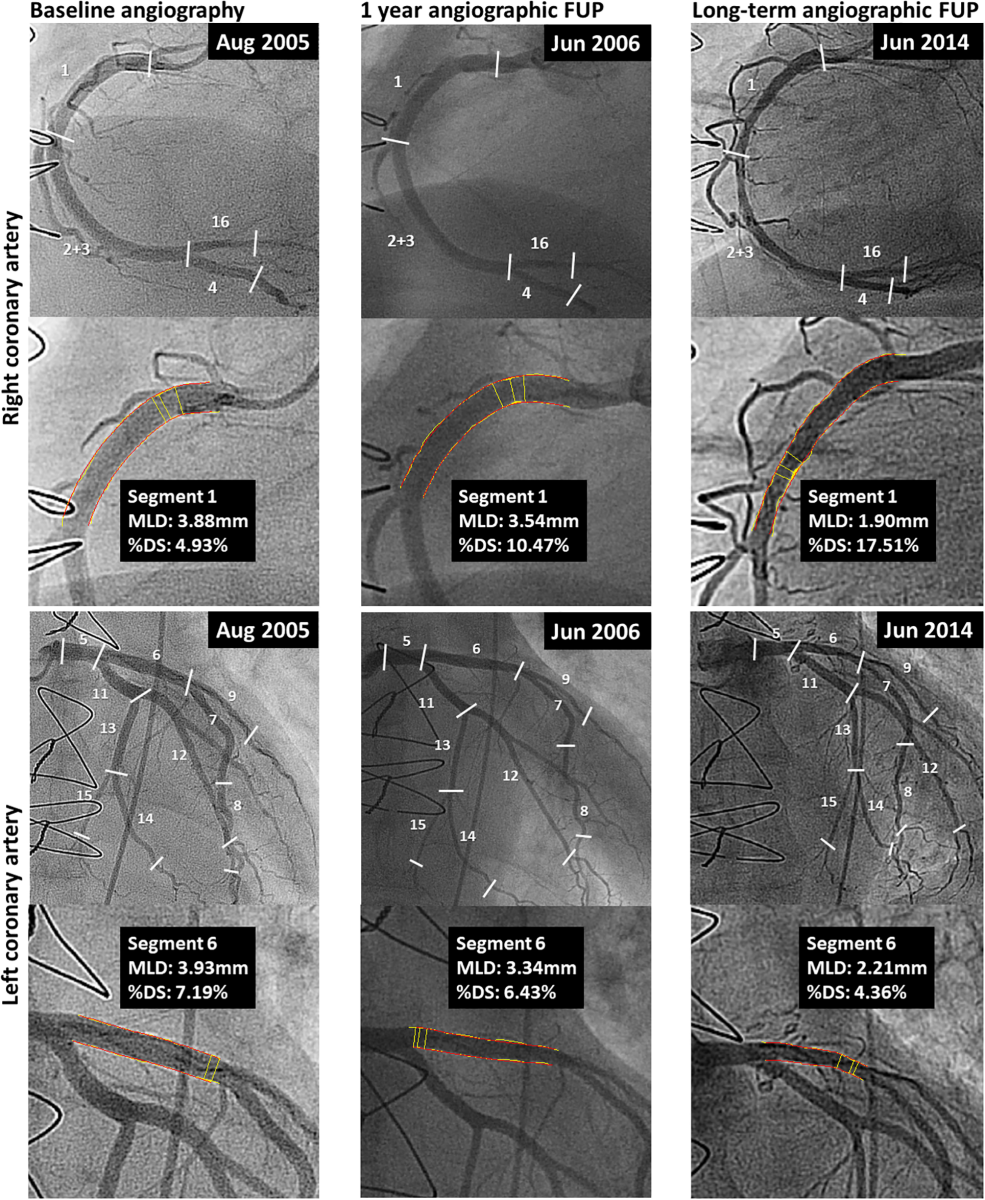
Serial 3-vessel quantitative coronary angiography analysis. This figure shows the serial quantitative coronary angiography analysis within matched regions of all coronary artery segments at baseline, at 1 year follow-up, and at latest available angiographic follow-up. Coronary artery segments were classified according to the modified AHA/ACC classification. MLD indicates minimal lumen diameter; %DS, percent diameter stenosis.

Standard biplane angiographic images were obtained after administration of intra-coronary nitroglycerin (100–300 μg) with a frame rate of 15/second. Each coronary segment was recorded in at least two orthogonal views. All angiographies were analyzed at the angiographic core laboratory at the University Hospital of Bern by two independent clinicians (CZ, KY), and a third clinician in case of disagreement (LR), who were blinded to any clinical data. All three major epicardial vessels including all side branches with a reference vessel diameter (RVD) of >1.5mm were assessed by QCA at baseline, at 1-year, and at long-term angiographic follow-up after HTx by using similar projections whenever possible. For this purpose, segments of the coronary vessels were divided into subsegments according to the modified American Heart Association/American College of Cardiology (AHA/ACC) [9] classification (Fig 2). QCA analysis was done by using the QangioXA version 7.3 (Medis Medical Imaging Systems, Leiden, the Netherlands). Minimal lumen diameter (MLD), RVD, segment length and maximal percent diameter stenosis [%DS, calculated by (1 –MLD/RVD) x 100] within the subsegments were assessed. In the case of interim revascularization, the revascularized vessel was analyzed prior to the revascularization, whereas the non-revascularized vessel segments were analyzed at the long-term angiographic follow-up. The change of all variables was derived for each segment as outcome (long-term angiographic follow-up and 1 year follow-up) minus outcome (1 year follow-up and baseline). The primary angiographic endpoint was mean change in MLD and %DS. Analysis was performed to determine the MLD and %DS change within the two groups (ISHLT-CAV_0_ vs. ISHLT-CAV_1-3_) 1 year after HTx and at long-term angiographic follow-up.

### Immunosuppression therapy and rejection score

The induction immunosuppressive therapy consisted of azathioprine 5 mg/kg and 1000 mg of methylprednisolone. Antithymocyte globulin (4–5 mg/kg) therapy was introduced within 12 hours post-transplant and thereafter tailored by CD3 cell count. A calcineurin inhibitor (CNI) based immunosuppressive therapy was initiated as a standard maintenance therapy. In case of progressive impairment of renal function, everolimus (ERL) was initiated already within the first month after HTx. Other reasons for switching to ERL were progressive CAV and skin malignancy or side effects. Switching to ERL therapy was a decision that was left to the discretion of the experienced transplant cardiologist. The general targeted ERL trough concentration (*C*_*0*_) was 6–8 ng/mL and 8–14 ng/mL for tacrolimus (TAC) with a concomitant therapy of prednisone and mycophenolate mofetil (MMF) or azathioprine. Endomyocardial biopsy (EMB) specimens were graded according to the 2005 ISHLT classification for acute cellular rejection (ACR) as 0R = 0, 1R = 1, 2R = 2, and 3R = 3 [13]. Severe Total Rejection Score (TRS) was defined as number of ACR ≥ 2R divided by the total numbers of biopsies. Antibody-mediated rejection (AMR) was diagnosed in the EMB specimens according to standardized histopathologic signs of AMR and, if indicated, with immunohistochemistry to confirm AMR. No immunohistochemistry positive EMBs or allograft rejections with hemodynamic compromise were observed.

### Clinical and demographic data

Clinical and angiographic data were retrieved by a retrospective review of all patient charts or database. The cardiovascular risk factors were monitored according to local guidelines. Hypertension was defined as systolic blood pressure ≥ 140 mmHg and/or diastolic blood pressure ≥ 90 mmHg. Hypercholesterolemia was defined as total cholesterol ≥ 5.2 mmol/L. MDRD (Modification of Diet in Renal Disease) glomerular filtration rate (shown as GFR; creatinine shown as Cr) was calculated by using the following formula: GFR = 186 x Serum-Cr -1.154 x age -0.203 x (0.742 if patient was female).

Acetylsalicyclic acid (ASA) therapy was continued after HTx in most patients with previous CAD or as indicated after percutaneous coronary intervention (PCI). After HTx, all patients received statin therapy. Ezetimibe was added in case of insufficient lipid lowering effect. In case of high-risk cytomegalovirus (CMV) serologic status (*i*.*e*., donor positive and recipient negative), HTx recipients received ganciclovir or valganciclovir CMV prophylaxis adapted to individual kidney function for 6 months. Pre-emptive CMV treatment was given in all other patients throughout the follow-up period.

### Statistical analysis

Continuous variables for patient-level data are expressed as median with interquartile range or mean ± standard deviation (SD). Categorical variables are expressed as number and percentage. Patients were stratified into two groups according to the presence or absence of ISHLT-CAV at 1 year and at latest angiographic follow-up. Comparisons between the groups regarding risk factors of CAV were performed by using the χ^2^ test. All hypotheses tested were 2-tailed and a *p*-value <0.05 was considered statistically significant.

QCA outcomes were recorded for segments per patient at baseline, at 1 year, and at long-term angiographic follow-up. For each segment, we computed the absolute change from baseline to 1-year, from baseline to long-term angiographic follow-up, and from 1-year to long-term angiographic follow-up. Patient-level outcomes and their changes were generated by using generalized linear mixed effect model. Wilcoxon rank sum test was used to compare the changes of parameters between baseline, 1 year, and long-term angiographic follow-up. To assess the subsegment-level data of the QCA analysis and its associations of all clinical variables, including the ISHLT-CAV grading score, a linear regression analysis was used that took into account a random effect of patient-level data.

Statistical analyses were performed with the use of R version 3.2.3 (R Foundation for Statistical Computing).

## Results

### Baseline characteristics

The donor and recipient characteristics of the study cohort are summarized in Table 1. The study population consisted of 77 patients (57 males), with a mean recipient age at HTx of 46.6 (±15.7) years. ERL-based therapy was administered in 41 (53%) and TAC-based therapy in 36 (47%) patients. The leading cause of HTx was dilated cardiomyopathy (n = 36; 47%), followed by ischemic cardiomyopathy (n = 27; 35%). During follow-up, 14 patients underwent PCI. Thirteen underwent PCI due to CAV and 1 patient due to fistula from the left anterior descending artery to the right ventricle. No patient had echocardiographic allograft dysfunction (defined as LVEF ≤ 45% with the presence of regional wall motion abnormalities) at latest follow-up.

**Table 1:** Baseline clinical characteristics.

	HTx recipients (n = 77)
Recipient age at transplantation (years)	46.6 ± 15.7
Donor age (years)	43.4 ± 13.2
Recipient (male)	57 (74)
Primary cause of HTx	
Dilated cardiomyopathy	36 (47)
Ischemic cardiomyopathy	27 (35)
Arrhythmogenic right ventricular cardiomyopathy	5 (6)
Hypertrophic cardiomyopathy	2 (3)
Valvular heart disease	2 (3)
Other causes*	5 (6)
Cardiac risk factors	
BMI (kg/m^2^)	24.6 ± 4.7
Hypertension	30 (39)
Systolic blood pressure (mmHg)	130 ± 17
Diastolic blood pressure (mmHg)	78 ± 12
HbA1c	5.8 ± 0.7
Active smoker	9 (12)
eGFR (mL/min)	65.2 ± 27.3
CMV serology, donor positiv/recipient negativ	21 (27)
CMV infection	29 (38)
Severe TRS ≥ 2R	0.040 ± 0.052
Lipids	
Hypercholesterolemia	37 (48)
Total cholesterol (mmol/L)	5.3 ± 1.5
HDL cholesterol (mmol/L)	1.6 ± 0.5
LDL cholesterol (mmol/L)	3.2 ± 1.2
Triglycerides (mmol/L)	2.2 ± 1.5
Medication	
ACEi / ARB	54 (70)
Beta Blocker	21 (27)
Calcium channel blocker	24 (31)
Aspirin or Clopidogrel	51 (66)
Statin	77 (100)
Ezetimibe	19 (25)
Immunosuppressive Regimes	
Everolimus, MMF, Prednisone	24 (31)
Everolimus, MMF	13 (17)
Everolimus, Prednisone	3 (4)
Everolimus, AZA, Prednisone	1 (1)
Tacrolimus, MMF	18 (23)
Tacrolimus, MMF, Prednisone	12 (16)
Tacrolimus, Prednisone	4 (5)
Tacrolimus, AZA	2 (3)
Echocardiography	
LVEF (%)	62 ± 6

Categorical variables are shown as number (%). Continuous variables are shown as mean ± SD

HTx, heart transplantation; BMI, body mass index; eGFR, estimated glomerular filtration rate; CMV, cytomegalovirus; TRS, total rejection score; HDL, high-density lipoprotein; LDL, low-density lipoprotein; ACEi, angiotensin-converting enzyme inhibitor; ARB, angiotensin receptor blocker; MMF, Mycophenolate mofetil; AZA, Azathioprin; LVEF, left ventricular ejection fraction

*Other causes: congenital heart disease (1 patient), giant cell myocarditis (1 patient), Naxos-Syndrome (1 patient), myofibrillar myopathy (1 patient), biventricular heart failure of unknown etiology (1 patient)

### Serial 3-vessel quantitative coronary angiography analyses

**Fig 3:**
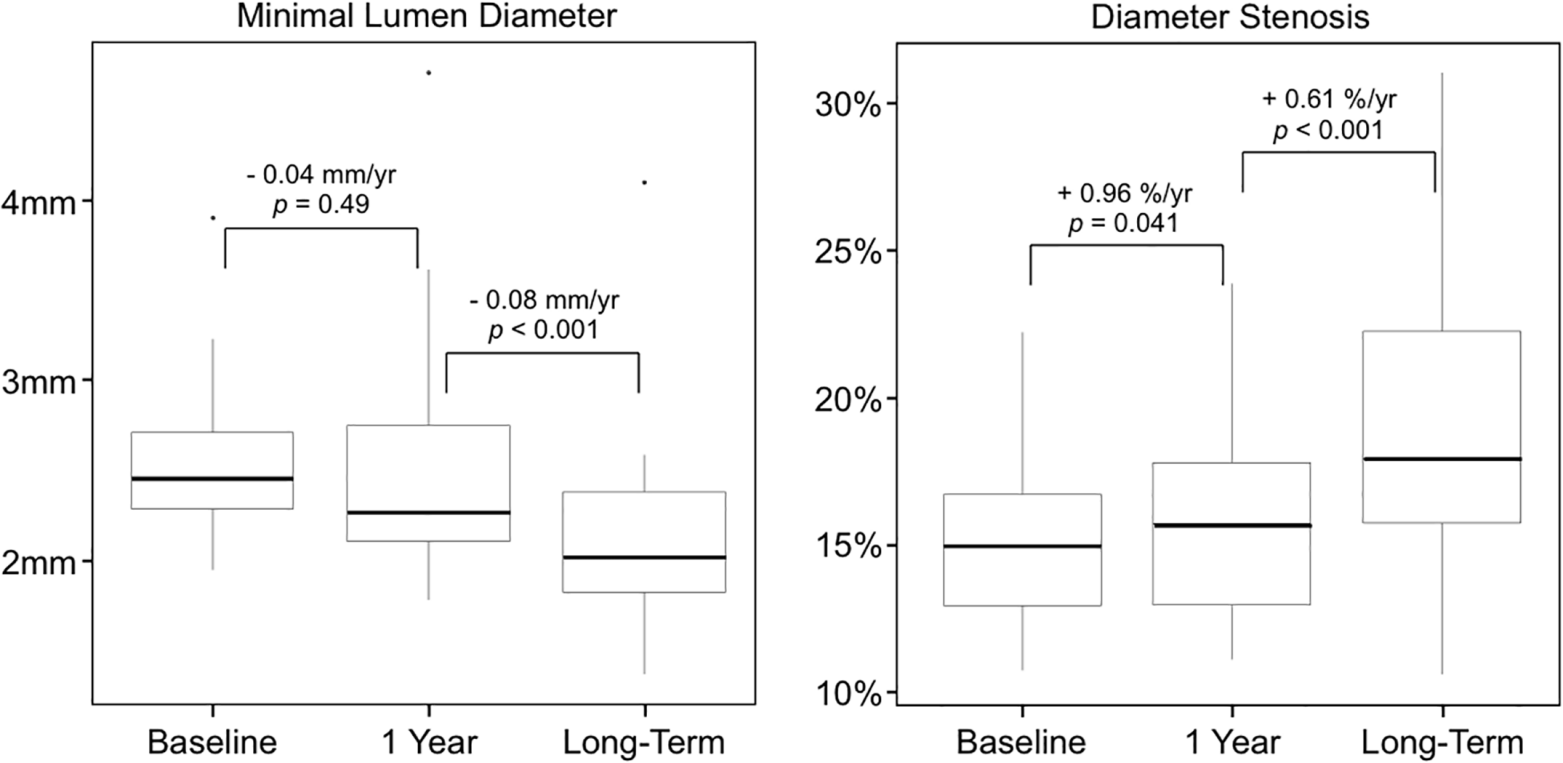
Serial 3-vessel quantitative coronary angiography analysis. Box-plot representation of minimal lumen diameter and maximal percent diameter stenosis at baseline (median 0.14 years [0.12 to 0.16], at 1 year (median 1.17 years [1.14 to 1.20]) and at long-term angiographic follow-up (median 8.61 years [8.24 to 8.99]) after HTx. Lower and upper box edges are the quartiles and thick line is the median.

A total of 520 native coronary artery segments (12.7 segments per patient) at baseline (median 0.14 [0.12 to 0.16] years after HTx) were matched with the corresponding segments at 1 year follow-up (median 1.17 [1.14 to 1.20] years after HTx) and at long-term angiographic follow-up (median 8.61 [8.24 to 8.99] years after HTx). Fig 3 shows the overall mean change of MLD and %DS from baseline to 1 year follow-up, and from 1 year follow-up to long-term angiographic follow-up. MLD decreased non-significantly from baseline to 1 year follow-up (Δ-0.04 mm/year [95% CI -0.17 to -0.08], *p* = 0.49), and significantly from 1 year follow-up to long-term angiographic follow-up (Δ-0.08 mm/year [95% CI -0.11 to -0.05], *p* < 0.001). %DS increased significantly from baseline to 1 year follow-up (Δ+0.96%/year [95% CI 0.04 to 1.88], *p* = 0.041), and from 1 year follow-up to long-term angiographic follow-up (Δ+0.61%/year [95% CI 0.33 to 0.88], *p* < 0.001).

**Fig 4:**
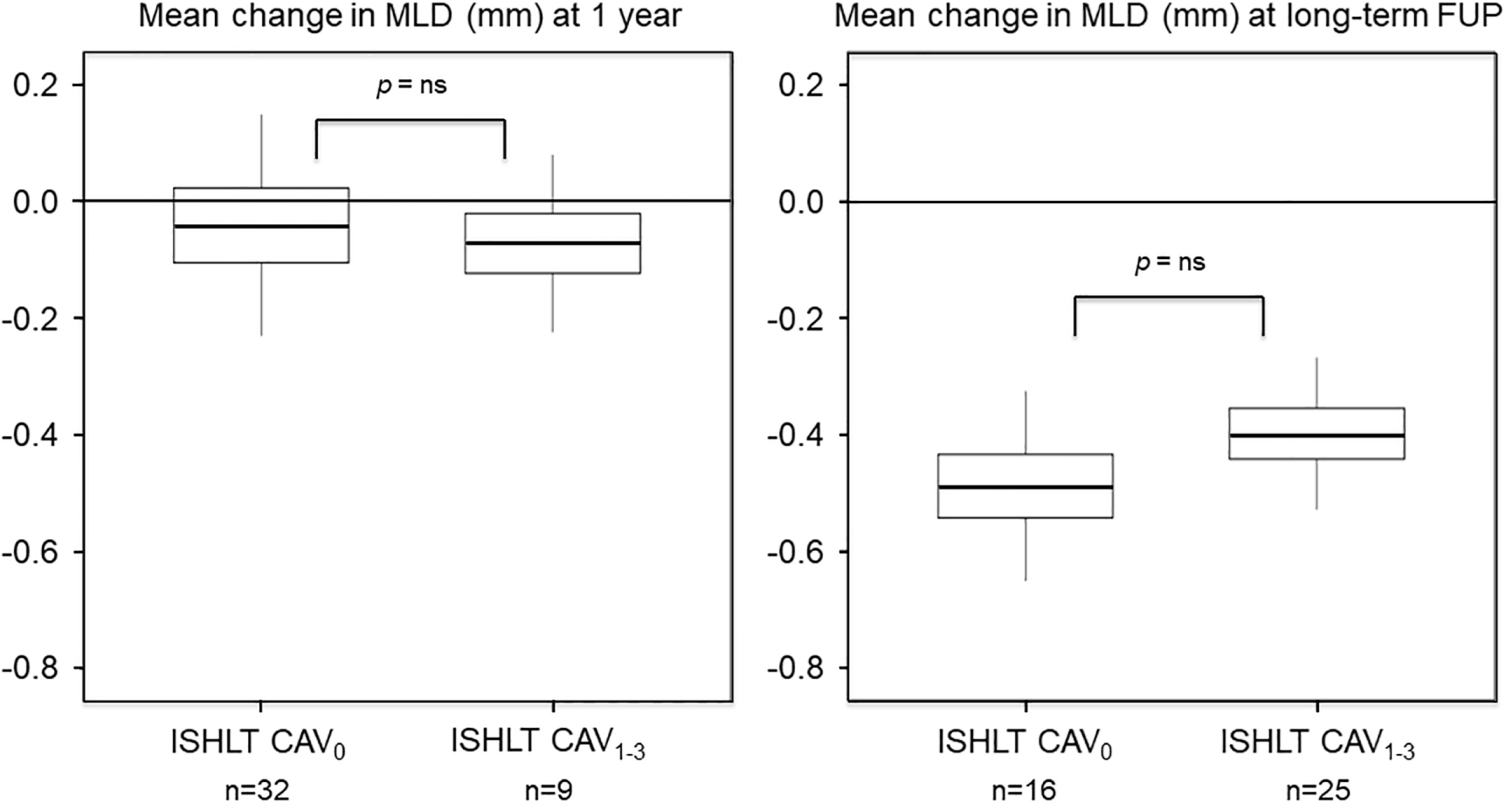
Quantitative angiographic analysis. Box-plot representation of the per-patient mean angiographic change in minimal lumen diameter (minimal lumen diameter 1 year—minimal lumen diameter baseline and minimal lumen diameter latest angiographic follow-up—minimal lumen diameter baseline) from coronary artery segments that were serially assessed and matched. The analysis is stratified according to absence (n = 16) or presence (n = 25) of ISHLT-CAV at 1 year, and at latest angiographic follow-up (median 8.61 years [8.24 to 8.99]). Lower and upper box edges are the quartiles and thick line is the median. A horizontal reference line at change = 0 is drawn.

Table 2 represents the serial 3-vessel QCA results between the ISHLT-CAV groups. On coronary angiography at 1 year, 32 patients (78%) were graded as ISHLT-CAV_0_, whereas 9 patients (22%) were graded as ISHLT-CAV_1-3_ [ISHLT CAV_1_, n = 9 (100%); ISHLT-CAV_2_, n = 0 (0%); ISHLT-CAV_3_, n = 0 (0%)]. At long-term angiographic follow-up, 16 patients (39%) were graded as ISHLT-CAV_0_, whereas 25 patients (61%) were graded as ISHLT-CAV_1-3_ [ISHLT-CAV_1_, n = 18 (72%); ISHLT-CAV_2_, n = 7 (28%); ISHLT-CAV_3_, n = 0 (0%)]. A reduction in MLD was observed within both groups between baseline and 1 year follow-up, and between baseline and long-term angiographic follow-up. The reduction in MLD between the two groups did not differ at 1 year (ISHLT-CAV_0_: median -0.04 mm [IQR -0.10 to 0.02] vs. ISHLT-CAV_1-3_: median -0.07 mm [IQR -0.12 to -0.02], *p* = 0.8) and during long-term angiographic follow-up (ISHLT-CAV_0_: median -0.49 mm [IQR -0.54 to -0.43] vs. ISHLT-CAV_1-3_: median -0.40 mm [IQR -0.44 to -0.35], *p* = 0.4) (Fig 4). Patients who developed ISHLT-CAV_1-3_ during long-term follow-up showed a significantly higher baseline %DS (ISHLT-CAV_0_: median 13.6% [IQR 13.2 to 14.1] vs. ISHLT-CAV_1-3_: median 15.9% [IQR 15.6 to 16.3], *p* = 0.01), and at long-term angiographic follow-up %DS (ISHLT-CAV_0_: median 15.9% [IQR 15.3 to 16.6] vs. ISHLT-CAV_1-3_: median 21.0% [20.5 to 21.5], *p* < 0.001). The increase in %DS between baseline and long-term angiographic follow-up was significantly higher in the ISHLT-CAV_1-3_ group (ISHLT-CAV_0_: median 2.3% [IQR 1.7 to 2.9] vs. ISHLT-CAV_1-3_: median 5.1% [IQR 4.6 to 5.5], *p* = 0.02).

**Table 2:** QCA analysis of serial angiography at baseline, 1 year and long-term follow-up after heart transplantation.

	All patients	At 1 year		*p*-value	At long-term follow-up		*p*-value
		ISHLT-CAV_0_	ISHLT-CAV_1-3_		ISHLT-CAV_0_	ISHLT-CAV_1-3_	
Number of patients	41	32	9		16	25	
Number of segments	520	408	112		204	316	
Number of segments per patient	12.7	12.8	12.4		12.8	12.6	
Reference Vessel Diameter (mm)							
Baseline	3.10 [3.06 to 3.15]	3.10 [3.05 to 3.15]	3.12 [3.02 to 3.22]	0.91	3.13 [3.06 to 3.21]	3.09 [3.02 to 3.15]	0.74
1-year	3.04 [2.97 to 3.12]	2.97 [2.89 to 3.06]	3.29 [3.13 to 3.44]	0.26	3.09 [2.97 to 3.21]	3.01 [2.91 to 3.10]	0.72
Latest FUP	2.67 [2.61 to 2.72]	2.65 [2.58 to 2.71]	2.73 [2.61 to 2.85]	0.67	2.58 [2.50 to 2.67]	2.72 [2.65 to 2.79]	0.43
Segment Length (mm)							
Baseline	36.1 [35.4 to 36.9]	36.0 [35.1 to 36.8]	36.6 [35.0 to 38.3]	0.81	34.9 [33.7 to 36.1]	36.9 [35.9 to 37.8]	0.41
1-year	36.7 [36.0 to 37.4]	36.1 [35.3 to 36.9]	38.8 [37.3 to 40.3]	0.31	34.6 [33.5 to 35.7]	38.0 [37.1 to 38.9]	0.12
Latest FUP	33.7 [33.1 to 34.3]	33.6 [33.0 to 34.3]	33.8 [32.5 to 35.1]	0.93	31.5 [30.6 to 32.4]	35.1 [34.3 to 35.8]	0.06
Total Segment Length (mm)							
Baseline	456.4 [446.7 to 466.1]	456.7 [445.6 to 467.9]	455.1 [434.1 to 476.1]	0.96	444.9 [429.2 to 460.5]	463.8 [451.2 to 476.3]	0.53
1-year	463.7 [454.8 to 472.7]	459.0 [448.8 to 469.2]	480.5 [461.2 to 499.8]	0.51	441.2 [427.0 to 455.4]	478.1 [466.8 to 489.5]	0.17
Latest FUP	426.4 [418.1 to 434.7]	428.0 [418.5 to 437.5]	420.6 [402.6 to 438.5]	0.8	401.6 [388.6 to 414.7]	442.2 [431.8 to 452.6]	0.11
Minimal Lumen Diameter (mm)							
Baseline	2.53 [2.49 to 2.57]	2.55 [2.50 to 2.59]	2.48 [2.39 to 2.56]	0.64	2.61 [2.54 to 2.67]	2.48 [2.43 to 2.54]	0.34
1-year	2.47 [2.41 to 2.53]	2.44 [2.37 to 2.51]	2.59 [2.46 to 2.71]	0.5	2.56 [2.47 to 2.66]	2.41 [2.34 to 2.49]	0.41
Latest FUP	2.10 [2.05 to 2.14]	2.09 [2.04 to 2.15]	2.11 [2.01 to 2.21]	0.94	2.11 [2.04 to 2.19]	2.09 [2.03 to 2.15]	0.87
Δ* in MLD (1 year-BL)	-0.06 [-0.10 to -0.02]	-0.11 [-0.15 to -0.06]	0.10 [0.02 to 0.19]	0.15	-0.04 [-0.10 to 0.02]	-0.07 [-0.12 to -0.02]	0.8
Δ* in MLD (FUP-BL)	-0.43 [-0.47 to -0.40]	-0.45 [-0.49 to -0.41]	-0.38 [-0.45 to -0.30]	0.56	-0.49 [-0.54 to -0.43]	-0.40 [-0.44 to -0.35]	0.4
Diameter Stenosis (%)							
Baseline	15.0 [14.7 to 15.3]	14.7 [14.4 to 15.0]	16.3 [15.7 to 17.0]	0.13	13.6 [13.2 to 14.1]	15.9 [15.6 to 16.3]	0.01
1-year	16.0 [15.67 to 16.4]	15.6 [15.2 to 16.0]	17.6 [16.9 to 18.4]	0.12	15.1 [14.5 to 15.6]	16.7 [16.2 to 17.1]	0.16
Latest FUP	19.0 [18.6 to 19.5]	18.2 [17.7 to 18.8]	21.9 [20.9 to 22.9]	0.04	15.9 [15.3 to 16.6]	21.0 [20.5 to 21.5]	<0.01
Δ* in %DS (1 year-BL)	1.0 [0.7 to 1.3]	0.9 [0.6 to 1.3]	1.3 [0.7 to 2.0]	0.72	1.4 [1.0 to 1.9]	0.7 [0.3 to 1.1]	0.43
Δ* in %DS (FUP-BL)	4.0 [3.6 to 4.4]	3.6 [3.1 to 4.0]	5.6 [4.8 to 6.4]	0.15	2.3 [1.7 to 2.9]	5.1 [4.6 to 5.5]	0.02

Patient-level outcomes derived as the median (mm) and interquartile range. P-values using generalized linear mixed effect model taking random effects of patient-level data into account. QCA indicates quantitative coronary angiography; BL, baseline; FUP, follow-up; MLD, minimal lumen diameter; %DS, percent diameter stenosis; Δ, difference;

*Change was derived at the level of segments.

### Cardiac allograft vasculopathy risk factors

Table 3 shows baseline clinical characteristics according to the presence or absence of ISHLT-CAV_1-3_ at latest angiographic follow-up. On latest coronary angiography, 39 patients (51%) were graded as ISHLT-CAV_0_, whereas 38 patients (49%) were graded as ISHLT-CAV_1-3_ [CAV_1_, n = 25 (66%); CAV_2_, n = 12 (32%); CAV_3_, n = 1 (3%)]. ISHLT- CAV_1-3_ was significantly associated with eight factors: higher recipient age (45.0 years [26.0–54.0] vs. 55.5 years [42.5–61.5]; *p* = 0.01); higher donor age (38.5 years [31.0–49.7] vs. 49.0 years [39.0–57.5]; *p* = 0.009); CAD before HTx (9 (23%) vs. 18 (47%); *p* = 0.046); lower eGFR (70.2 ml/min [52.1–87.4] vs. 54.8 ml/min [39.4–66.8]; *p* = 0.005); higher LDL cholesterol (2.5 mmol/l [2.1–3.7] vs. 3.2 mmol/l [2.5–3.9]; *p* = 0.08); higher triglyceride (1.5 mmol/l [1.1–2.0] vs. 2.3 mmol/l [1.3–3.8]; *p* = 0.01); severe TRS ≥ 2 (0.00 [0.00–0.05] vs. 0.05 [0.00–0.09]; *p* = 0.005); and male donor (24 (62%) vs. 32 (84%); *p* = 0.07). Higher max %DS was significantly associated with five factors: higher recipient age (48.0 years [22.0–56.5] vs. 53.0 years [41.2–60.0]; *p* = 0.04); higher HbA1c (5.5 [5.3–5.8] vs. 5.8 [5.6–6.1]; *p* = 0.004); lower eGFR (68.2 ml/min [56.9–89.2] vs. 53.8 ml/min [39.4–69.3]; *p* = 0.002); higher LDL cholesterol 2.5 mmol/l [2.1–3.6] vs. 3.2 mmol/l [2.5–4.0]; *p* = 0.09); and severe TRS ≥ 2 (0.00 [0.00–0.06] vs. 0.04 [0.00–0.09]; *p* = 0.03). There were no significant differences between the two groups regarding the adherence to immunosuppressive medications and cardiovascular medication including ERL, TAC, MMF, azathioprine, prednisone, ACE inhibitor/ARB, statin, and ezetimibe.

**Table 3:** Baseline clinical characteristics in patients with and without ISHLT-CAV at latest follow-up.

	ISHLT-CAV_0_ at latest follow-up n = 39	ISHLT-CAV_1-3_ at latest follow-up n = 38	*p*-value	Lower max %DS n = 39	Higher max %DS n = 38	*p*-value
Recipient profile						
Age (years)	45.0 [26.0–54.0]	55.5 [42.5–61.5]	0.01	48.0 [22.0–56.5]	53.0 [41.2–60.0]	0.04
Male	28 (72)	29 (76)	0.85	27 (69)	30 (79)	0.48
Body mass index, kg/m^2^	24.2 [22.5–26.4]	24.1 [20.6–27.9]	0.83	23.81 [21.5–26.2]	24.3 [21.6–28.1]	0.29
CAD before HTx	9 (23)	18 (47)	0.046	11 (28)	16 (42)	0.3
Hypertension	15 (38)	15 (39)	1	13 (33)	17 (45)	0.43
HbA1c (%)	5.6 [5.4–5.8]	5.7 [5.4–6.1]	0.12	5.5 [5.3–5.8]	5.8 [5.6–6.1]	0.004
Active Smoker	5 (13)	4 (11)	1	4 (10)	5 (13)	0.97
eGFR (ml/min)	70.2 [52.1–87.4]	54.8 [39.4–66.8]	0.005	68.2 [56.9–89.2]	53.8 [39.4–69.3]	0.002
Hypercholesteremia	16 (41)	21 (55)	0.31	18 (46)	19 (50)	0.91
Total cholesterol (mmol/L)	4.5 [4.0–5.8]	5.2 [4.5–6.2]	0.13	4.9 [4.0–5.8]	5.2 [4.5–6.2]	0.19
HDL cholesterol (mmol/L)	1.6 [1.2–2.1]	1.5 [1.2–1.8]	0.49	1.5 [1.2–1.9]	1.6 [1.3–1.9]	0.57
LDL cholesterol (mmol/L)	2.5 [2.1–3.7]	3.2 [2.5–3.9]	0.08	2.5 [2.1–3.6]	3.2 [2.5–4.0]	0.09
Triglyceride (mmol/L)	1.5 [1.1–2.0]	2.3 [1.3–3.8]	0.01	1.8 [1.1–2.4]	2.0 [1.3–2.8]	0.52
CMV, donor pos./ recipient neg.	8 (21)	13 (34)	0.27	10 (25)	11 (29)	0.94
CMV infection	12 (31)	17 (45)	0.3	12 (31)	17 (45)	0.3
Severe TRS ≥ 2R	0.00 [0.00–0.05]	0.05 [0.00–0.09]	0.005	0.00 [0.00–0.06]	0.04 [0.00–0.09]	0.03
LVEF-FUP (%)	65.0 [60.0–65.0]	60.0 [60.0–65.0]	0.07	65.0 [60.0–65.0]	60.0 [60.0–65.0]	0.19
LVEDP (mmHg)	12.5 [8.5–17.0]	11.5 [6.0–15.0]	0.25	13.0 [8.0–17.5]	11.0 [6.0–14.0]	0.13
Donor profile						
Age (years)	38.5 [31.0–49.7]	49.0 [39.0–57.5]	0.009	42.0 [33.0–52.8]	48.0 [36.2–55.5]	0.16
Male	24 (62)	32 (84)	0.07	25 (64)	31 (82)	0.19

Values shown are median [lower to upper quartile] or number (%), *p*<0.05 was considered statistically significant; CAD, coronary artery disease; eGFR, estimated glomerular filtration rate; HDL, high-density lipoproteins; LDL, low-density lipoproteins; MLD, minimal lumen diameter; RVD, reference vessel diameter; CMV, cytomegalovirus; TRS, total rejection score; LVEF, left ventricular ejection fraction; FUP, follow-up, LVEDP, left ventricular end diastolic pressure.

## Discussion

The four main findings of this study can be summarized as follows. First, patients without visual signs of CAV during follow-up showed similar MLD loss compared with ISHLT-CAV_1-3_ patients. Secondly, MLD loss between baseline and 1-year after HTx did not predict CAV progression beyond 1 year. Thirdly, baseline %DS was higher in patients who were developing ISHLT-CAV_1-3_ at latest angiographic follow-up. Fourth, risk factors of CAV according to the ISHLT-CAV classification were in line with the risk factors associated with the QCA analyses.

### Use of serial three-vessel QCA to assess cardiac allograft vasculopathy progression

The present study made it possible to assess short- and long-term progression of CAV with the current standard of visual ISHLT-CAV classification and the serial 3-vessel QCA analyses within all segments of the three coronary arteries. The advantage of using QCA is that it detects subtle changes of luminal narrowing. IVUS studies have shown that approximately 80% of patients already show a thickening of the coronary intima within the first year after HTx [9]. Of note, most of those patients generate the intimal thickening without angiographical signs of CAV. Importantly, the most rapid rate of intimal thickening occurs during the first year after HTx, followed by a slower rate of intimal thickening. Furthermore, a rapid progression of maximal intimal thickness of >0.5 mm during the first year after HTx has been shown to be predictive of angiographic CAV after five years [14] and of increased risk of all-cause mortality and myocardial infarction [10]. Due to a lack of standardized predictive value of lumen change between baseline and 1 year, such cut-off values are not available for QCA in the current analysis. In contrast to our expectation, the reduction in MLD from baseline to long-term angiographic follow-up did not differ between patients with ISHLT-CAV_0_ and ISHLT-CAV_1-3_ (ISHLT-CAV_0_: median -0.49 mm [IQR -0.54 to -0.43] vs. ISHLT-CAV_1-3_: median -0.40 mm [IQR -0.44 to -0.35], *p* = 0.4). A failure in QCA to detect a significant lumen loss between baseline and 1 year, as an indirect indicator of the vessel wall thickening, might be influenced by an expansion of the external elastic membrane leading to a preservation of the luminal area without angiographic signs of CAV [15,16]. Hence, since angiography only depicts the lumen contour, QCA could not identify early intimal thickening by narrowing of the lumen area in patients who developed ISHLT-CAV_1-3_ during long-term follow-up. Furthermore, the similarity in the lumen loss between the two groups during long-term follow-up might be caused by a diffuse longitudinal and concentric narrowing of the coronary artery vessel wall that decreases MLD even though no angiographic signs of CAV were apparent. Furthermore, RVD decreased over time in a similar way like MLD, which indicates that the lumen narrowed in the coronary artery tree without visual plaque stenosis. This pathophysiological mechanism of CAV progression has been previously described in histopathology studies [17–19]. Hence, diffuse concentric intimal thickening and narrow points of constriction may be overlooked with coronary angiography, and applying the ISHLT classification might underestimate the CAV progression since it is mainly defined by stenotic plaques. As shown in our study, %DS expressed the vascular plaque stenosis in accordance to the ISHLT-CAV classification, which shows a significant difference in %DS at baseline and during long-term angiographic follow-up between the ISHLT-CAV groups.

Arora et.al. [7] demonstrated a significantly reduced CAV progression, and significantly less coronary intimal thickening, in patients treated with *de novo* everolimus-based therapy and early calcineurin inhibitor withdrawal, compared to the calcineurin inhibitor control group irrespective of donor disease in a matched IVUS examination at baseline and 12 months after HTx. In our cohort of 41 patients, 53% of the patients received everolimus-based and calcineurin inhibitor-free immunosuppressive therapy at latest angiographic follow-up, 42% of whom switched therapies within the first month after HTx. Hence, the use of potent immunosuppressive drugs in our cohort such as mycophenolate mofetil and everolimus might have decelerated CAV progression. In our cohort, including matched serial three vessel QCA analyses, 78% had no visual signs of CAV one year after HTx, a finding possibly influenced by the early everolimus therapy after HTx. However, during long-term angiographic follow-up there was no significant difference in the presence of CAV between the everolimus-based group compared with the tacrolimus-based group. Furthermore, the prevalence of CAV detected by coronary angiography at 1, 5, and 10 years after HTx is approximately 10–20%, 35–50%, and 50–60% [9], respectively. This finding is in line with our data, which report the prevalence of CAV at 1 year, and at a mean follow-up time of 9 years in 22% and 61% of patients, respectively. Of note, only 1 patient had visual sign of donor transmitted CAD at baseline.

### Risk factors of cardiac allograft vasculopathy

Several risk factors have been linked with CAV development including increasing donor age, donor history of hypertension, pre-transplant CAD, and cardiovascular risk factors [20]. To further validate the QCA method in our cohort, we assessed the risk factors of CAV with %DS and the ISHLT-CAV classification. Risk factors of CAV associated with maximal %DS and the ISHLT-CAV classification were similar, including recipient age, HbA1c, eGFR, and severe TRS ≥ 2R. Even though QCA is a more sensitive method than the visual assessment in identifying subtle changes of the coronary artery tree, it could not identify additional risk factors compared to the ISHLT-CAV classification. Surprisingly, the ISHLT-CAV classification additionally detected donor age, donor male, CAD before HTx, and triglycerides as CAV risk factors. Delgado et al. [21] also showed that donor age, CMV infection, and presence of cellular acute rejection ≥ 2R are predictors of CAV. In our study, CMV infection was not associated with CAV, which is in conflict with recent large cohort studies [21,22]. In a review by Braga et al. [23] the inconsistency of the CAV risk factors was suggested to be influenced by different CAV assessment methods, classification, and observation time.

The results of our single-centre retrospective study have to be interpreted in the light of some limitations. First, the lack of serial angiographies resulted in a limited sample size. Second, patients with more side branches, but with a reference diameter >1.5mm, could have a lower MLD not reflective of CAV. Nevertheless, baseline MLD between the two groups showed no significant difference in MLD and RVD, thus representing comparable groups. Furthermore, there was no significant difference between the two groups regarding the measured segments per patient and segment length. Consequently, the results should be regarded as exploratory.

## Conclusions

Mean lumen diameter loss between baseline and 1-year after heart transplantation as assessed by QCA in all coronary artery segments did not predict CAV progression in the long-term. There was no difference in MLD loss over time within the coronary tree between ISHLT-CAV_0_ and ISHLT-CAV_1-3_ patients as assessed by QCA, suggesting a similar progression of CAV independently of the ISHLT-CAV classification.

## Supporting information

S1 Data (XLSX)

S2 Data (XLSX)
